# Vitamin D 25OH Deficiency and Mortality in Moderate to Severe COVID-19: A Multi-Center Prospective Observational Study

**DOI:** 10.3389/fnut.2022.934258

**Published:** 2022-07-05

**Authors:** Laura Bogliolo, Emanuele Cereda, Catherine Klersy, Ludovico De Stefano, Federica Lobascio, Sara Masi, Silvia Crotti, Serena Bugatti, Carlomaurizio Montecucco, Stefania Demontis, Annalisa Mascheroni, Nadia Cerutti, Alberto Malesci, Salvatore Corrao, Riccardo Caccialanza, The NUTRI-COVID19 Collaborative Working Group

**Affiliations:** ^1^Division of Rheumatology, Fondazione IRCCS Policlinico San Matteo, Pavia, Italy; ^2^Clinical Nutrition and Dietetics Unit, Fondazione IRCCS Policlinico San Matteo, Pavia, Italy; ^3^Clinical Epidemiology & Biostatistics Unit, Fondazione IRCCS Policlinico San Matteo, Pavia, Italy; ^4^Nutritional Unit, Giovanni Borea Civil Hospital, Sanremo, Italy; ^5^Clinical Nutrition and Dietetics Unit–ASST Melegnano e Martesana, Melegnano (Milano), Italy; ^6^Medicine and Dietetics Unit, ASST Pavia, Pavia, Italy; ^7^Division of Gastroenterology and Digestive Endoscopy, Department of Gastroenterology, Humanitas Research Hospital, Milano, Italy; ^8^Department of Health Promotion Sciences, Maternal and Infant Care, Internal Medicine and Medical Specialties (PROMISE), University of Palermo, Palermo, Italy; ^9^COVID Unit, Department of Internal Medicine, National Relevance and High Specialization Hospital Trust ARNAS Civico, Di Cristina, Benfratelli, Palermo, Italy

**Keywords:** COVID-19, vitamin D 25OH, hospitalized patients, mortality, propensity score (PS)

## Abstract

**Introduction:**

Several studies and meta-analyses suggested the role of vitamin D 25OH in preventing severe forms of coronavirus disease 2019 (COVID-19). However, the evidence on the clinical benefits of vitamin D 25OH adequacy in patients hospitalized for COVID-19 remain conflicting and speculative. We aimed to investigate the association between vitamin D 25OH serum levels and mortality in hospitalized patients with moderate to severe COVID-19.

**Method:**

This prospective observational multicentre study included 361 consecutive patients with moderate to severe COVID-19 admitted to the Italian hospitals involved in the NUTRI-COVID19 trial from March to August 2020. For each patient, serum vitamin D 25OH levels were assessed 48 h since admission and classified as deficient (<20 ng/mL) or adequate (≥20 ng/mL). We built a propensity score for low/adequate vitamin D 25OH levels to balance the clinical and demographic properties of the cohort, which resulted in 261 patients with good common support used for the survival analysis.

**Results:**

Two Hundred-seventy-seven (77%) of the 361 enrolled patients (207 [57%] males, median age 73 ± 15.6 years) had vitamin D 25OH deficiency. Fifty-two (20%) of the 261 matched patients died during the hospital stay, corresponding to a hazard ratio of 1.18 for vitamin D 25OH deficiency (95% confidence interval: 0.86–1.62; *p* = 0.29).

**Discussion:**

The prevalence of vitamin D 25OH deficiency was confirmed to be very high in hospitalized patients with COVID-19. The use of a propensity score demonstrate an absence of significant association between vitamin D deficiency and mortality in hospitalized patients.

## Introduction

Several observational studies and meta-analyses suggested the protective role of vitamin D 25OH in coronavirus disease 2019 (COVID-19) (1, 2), due to its immunomodulatory and anti-inflammatory properties and its ability to modulate endothelial functions (3, 4). However, the clinical benefits of vitamin D 25OH adequacy in patients hospitalized for COVID-19 remain conflicting and speculative (5–8). In a recent paper published by our group, we surprisingly observed that vitamin D 25OH levels were proportionally associated with mortality in patients with COVID-19 (9). However, the limited sample and the severity of the patients' clinical conditions could have influenced the observed results. Hence, we decided to increase the sample size to investigate further the association between vitamin D 25OH levels and mortality in patients hospitalized for moderate to severe COVID-19. For this purpose, we conducted the present prospective observational study, which included patients admitted to the Italian hospitals involved in the multicentric NUTRI-COVID19 trial (10).

## Methods

**Figure 1:**
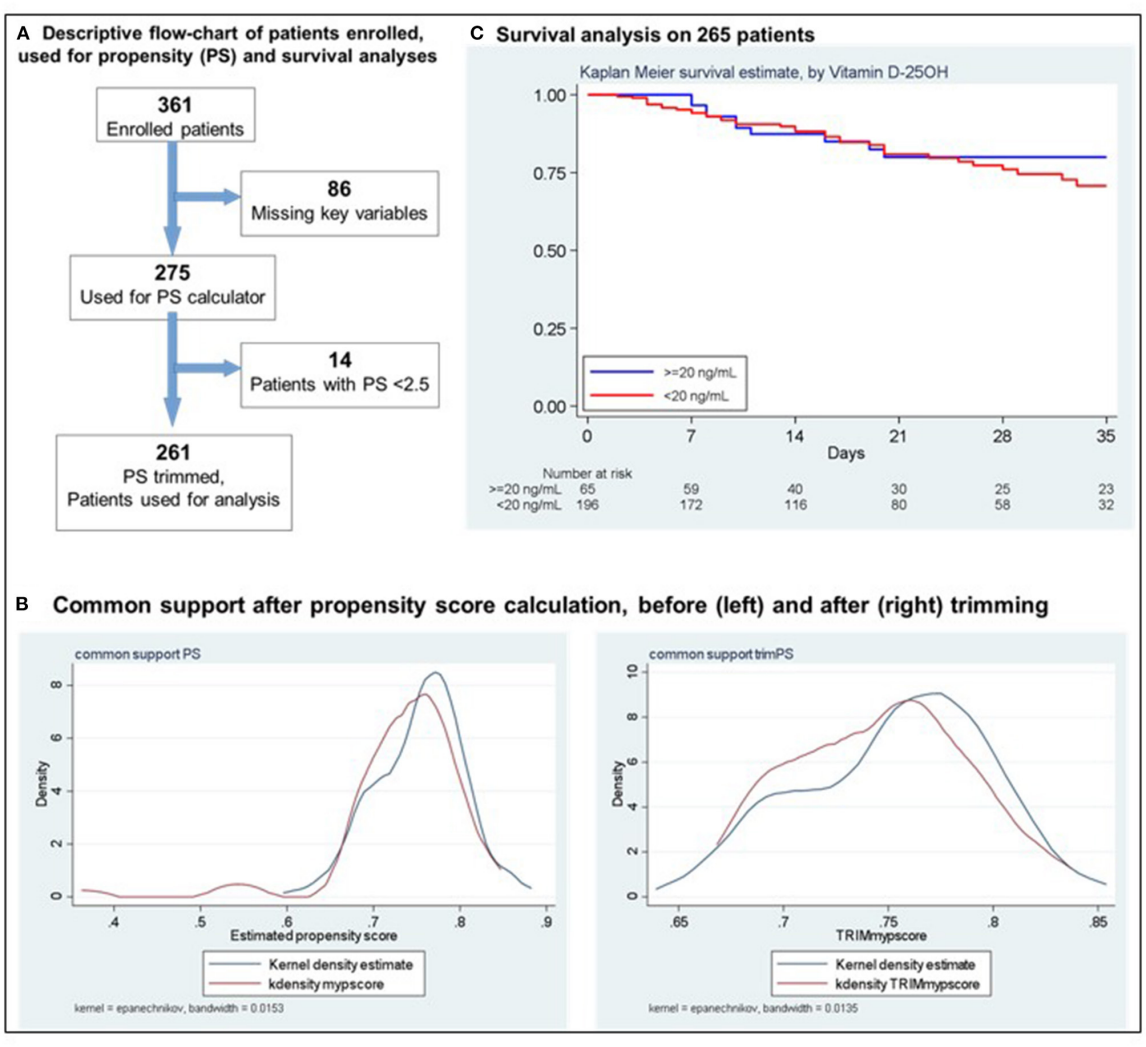
**(A)** Descriptive flow-chart of patients enrolled used for propensity (PS) and Survival analyses. **(B)** Common support after propensity score calculation, before (left) and after (right) trimming. **(C)** Survival analysis on 265 patients.

The multicentre cohort consisted of 361 consecutive COVID-19 patients (nasopharyngeal reverse transcriptase-polymerase chain reaction positive swab) admitted to six Italian hospitals between March and August 2020, not included in experimental treatment protocols. The local Institutional Ethics Committees approved the study, and written informed consent was obtained from every patient. Each patient was tested within 48 h since admission for serum vitamin D 25OH status [chemiluminescent immunoassay (Abbott Diagnostics, Lake Forest, IL, USA)] and, based on the results, was classified as adequate (≥20 ng/mL) or deficient (<20 ng/mL). Of the 361 hospitalized patients, complete data on key variables for mortality (age, sex, C-reactive protein [PCR], lactic acid dehydrogenase [LDH], body mass index [BMI], major comorbidities, and severe pneumonia) were available for 275 patients. Considering the significant clinical and laboratory differences between patients admitted to our and the other hospitals and the heterogeneity of the key variables in the enrolled patients, we fitted a Cox model to assess the association of vitamin D 25OH and in-hospital mortality, weighting the analysis by the inverse propensity score. This weight was derived from a propensity score for low/adequate vitamin D 25OH levels, including the key clinical and demographic properties. After trimming the upper and lower 2.5th percentiles of the score, the procedure yielded 261 patients with good common support (Figures 1A,B). All the analyses were performed using Stata 16 (StataCorp. College Station, TX, USA).

## Results

Two Hundred-seventy seven (77%) of the 361 enrolled patients (207 [57%] males, median age 73 ± 15.6 years) had vitamin D 25OH deficiency. No significant differences were observed in demographic and clinical features according to vitamin D 25OH status. In the 275 patients considered for the propensity score, a statistically significant difference in PCR values between those with adequate and deficient vitamin D 25OH serum levels [11.72 (1.52−8.12) vs. 9.64 (2.25–14.93), respectively; *p* = 0.02] was detected (Table 1). After a median follow-up of 20 days (95% confidence interval [CI]:10–33), in the propensity score-matched and trimmed group (*N* = 261), 52 patients died during the hospital stay. Thirteen had vitamin D 25OH adequate levels (mortality rate 5.3 per 100 per year, 95% CI: 3.1–9.6), 39 had vitamin D 25OH deficiency (mortality rate 6. per 100 per year, 95% CI: 4.8–8.9), which resulted in a hazard ratio [HR] of 1.18 (95% CI: 0.86–1.62; *p* = 0.29) (Figure 1C). A sensitivity analysis addressing severe deficiency (vitamin D 25OH <10 ng/mL, *p* = 0.64; <5 ng/mL, *p* = 0.14) yielded similar results. A further model using vitamin D 25OH on a continuous scale (linearity checked using fractional polynomials) confirmed the lack of association with the outcome (HR = 1.01, 95%CI 0.99–1.04; *p* = 029).

**Table 1:** Clinical and demographic characteristics of the enrolled patients.

	**All patients in the cohort** ***N*** **=** **361**			**Patients used for PS building** ***N*** **=** **275**		
**Feature**	**25(OH)Vitamin D≥20 ng/mL *N* = 84 (23%)**	**25(OH)Vitamin D <20 ng/mL *N* = 277 (77%)**	**P-value**	**25(OH)Vitamin D≥20 ng/mL *N* = 69 (25%)**	**25(OH)Vitamin D <20 ng/mL *N* = 206 (75%)**	**P-value**
Male, *N* (%)	41 (480.8)	166 (590.9)	0.04	36 (230.4)	118 (760.6)	0.27
Age, Median (IQR)	710.7 (660.5–810.5)	690.5 (590.0–820.0)	0.67	710.7 (640.0–810.0)	700.3 (600.0–840.0)	0.77
Body mass index (kg/m^2^), Median (IQR)	240.45 (220.7–270.3)	250.5 (220.5–280.1)	0.21	240.3 (220.7– 270.3)	250.2 (220.2–270.7)	0.38
COPD, *N* (%)	13 (150.5)	34 (120.3)	0.28	11 (150.9)	26 (120.6)	0.30
Diabetes, *N* (%)	21 (25)	78 (280.3)	0.33	18 (260.1)	63 (300.6)	0.29
Hypertension, *N* (%)	49 (580.3)	172 (620.3)	0.29	40 (58)	134 (65)	0.18
Ischemic heart disease, *N* (%)	23 (270.4)	86 (310.2)	0.30	19 (270.5)	65 (310.5)	0.32
Cancer, *N* (%)	17 (200.2)	37 (130.4)	0.09	13 (180.8)	31 (150.1)	0.29
Chronic kidney disease, *N* (%)	14 (160.7)	48 (170.3)	0.51	11 (150.9)	42 (200.4)	0.26
Number of comorbidities, Median (IQR)	2 (1–3)	2 (1–3)	0.64	2 (1–3)	2 (1–3)	0.69
Lactate dehydrogenase (U/L), Median (IQR)	338 (203–345)	305 (210–369)	0.67	3360.14 (2030.−343)	3030.93 (261–359)	0.54
C–reactive protein (mg/dL), Median (IQR)	110.04 (10.71–90.29)	80.98 (10.88–140.50)	0.07	110.72 (10.52– 80.12)	90.64 (20.25– 140.93)	0.02
Severe pneumonia ^a^, *N*(%)	41 (490.4)	143 (510.8)	0.39	32 (460.4)	96 (460.6)	0.54
Pavia Hospital	30 (350.7)	99 (350.7)	0.55	29 (42)	94 (450.6)	0.35
Other Hospitals	54 (640.3)	178 (640.2)	0.55	40 (570.9)	112 (540.4)	0.35

***COPD**, chronic obstructive pulmonary disease; **c–PAP** continuous Positive Airway Pressure*;

a *According to the American Thoracic Society guidelines0. PS, propensity score0*.

## Discussion

This study confirms that vitamin D 25OH deficiency is prevalent in hospitalized COVID−19 patients, with close to 80% prevalence rates. However, this deficiency is not associated with increased mortality. Our results contrast with some other studies, such as a recent retrospective observational study from Saudi Arabia, which detected an association between severe deficiency and mortality (11). On the other hand, in line with our findings, a recent meta–analysis confirmed the absence of a correlation between vitamin D 25OH supplementation and clinical outcomes (admission to Intensive Care Units and mortality) in COVID−19 patients (12). Moreover, in a recent randomized trial conducted on 240 patients, a single dose (200.000 IU) of vitamin D 25OH did not significantly reduce in–hospital mortality compared to placebo (13); nor did small doses of 25OH vitamin D administered daily reduce mortality, although it shortened recovery symptoms (14). In the recent spread of articles attempting to correlate vitamin D 25OH levels with clinical outcomes in COVID−19, our choice to use a propensity score to limit confounding factors may have helped clarify this issue. Although vitamin D 25OH has an acknowledged immunomodulatory function which could reduce the risk of COVID−19 infection, it probably cannot limit its progression in hospitalized and severe patients, in whom the extensive activation of innate effectors and the ineffective adaptive T lymphocytes–mediated response may have a decisive role in determining the disease outcomes (15). Considering the high prevalence of vitamin D 25OH deficiency observed, in line with a recent and updated meta–analysis (16), our confirmatory results may reinforce the hypothesis that adequate vitamin D 25OH levels could be protective in reducing the risk of hospitalization for COVID−19, but not for the survival in hospitalized patients with moderate to severe disease. However, although our propensity score–based study design allowed us to perform a more robust analysis of a multivariable model, avoiding the risk of overfitting, it should be acknowledged that it could be associated with bias due to potential unmeasured confounders. It is also important to specify that no pre–admission vitamin D values were available. It is therefore difficult to establish whether the vitamin D deficiency depends on the infection or on an earlier status. Given the age of the population and the known epidemiological data on endemic vitamin D deficiency, as well as the relative stability of the 25–hydroxy form, a pre–existing deficit could be hypothesized. On the other hand, a sustained inflammatory burden–as the one characterizing COVID−19–could be responsible for a shorter half–life of vitamin–binding proteins and an increase in total body water, thus resulting in low serum concentrations (17). In light of these limitations, our results confirm that even though 25OH vitamin D may have a protective role in preventing severe COVID−19, its deficiency is not associated with increased mortality in hospitalized patients with moderate to severe disease.

## Data Availability Statement

The original contributions presented in the study are included in the article/supplementary material, further inquiries can be directed to the corresponding author.

## Ethics Statement

The studies involving human participants were reviewed and approved by Comitato Etico referente Area di Pavia, Fondazione IRCCS Policlinico San Matteo, Pavia. The patients/participants provided their written informed consent to participate in this study.

## Author Contributions

LB, EC, and RC had full access to all the data in the study and take responsibility for the integrity of the data and the accuracy of data analysis. RC is chief investigators and act as guarantors for this work. Concept and design: LB, CK, RC, and EC. Acquisition, analysis, or interpretation of data: RC, EC, LB, CK, LD, FL, SM, SC, SB, SD, AM, NC, AM, and SC. Drafting of the manuscript: LB, RC, EC, CK, and LD. Statistical analysis: CK. All authors contributed to the article and approved the submitted version.

## Funding

This study was supported in part by fundings from the IRCCS Policlinico San Matteo Foundation, Pavia, Italy.

## Conflict of Interest

The authors declare that the research was conducted in the absence of any commercial or financial relationships that could be construed as a potential conflict of interest.

## Publisher's Note

All claims expressed in this article are solely those of the authors and do not necessarily represent those of their affiliated organizations, or those of the publisher, the editors and the reviewers. Any product that may be evaluated in this article, or claim that may be made by its manufacturer, is not guaranteed or endorsed by the publisher.
